# Prognostic value of chronicity grading on renal outcomes in patients with IgA nephropathy

**DOI:** 10.3389/fmed.2022.952050

**Published:** 2022-08-24

**Authors:** Donghyuk Kang, Tae Hyun Ban, Ho Jun Chin, Hajeong Lee, Se Won Oh, Cheol Whee Park, Chul Woo Yang, Bum Soon Choi

**Affiliations:** ^1^Department of Nephrology and Hypertension, Korea University Guro Hospital, Korea University College of Medicine, Seoul, South Korea; ^2^Division of Nephrology, Department of Internal Medicine, Eunpyeong St. Mary's Hospital, The Catholic University College of Medicine, Seoul, South Korea; ^3^Division of Nephrology, Department of Internal Medicine, Seoul National University Bundang Hospital, Seoul National University College of Medicine, Seoul, South Korea; ^4^Division of Nephrology, Department of Internal Medicine, Seoul National University Hospital, Seoul National University College of Medicine, Seoul, South Korea; ^5^Division of Nephrology, Department of Internal Medicine, Korea University Anam Hospital, Korea University College of Medicine, Seoul, South Korea; ^6^Division of Nephrology, Department of Internal Medicine, Seoul St. Mary's Hospital, The Catholic University College of Medicine, Seoul, South Korea

**Keywords:** IgA nephropathy, end-stage renal disease, renal biopsy, pathology, glomerulosclerosis, interstitial fibrosis

## Abstract

Many studies have shown that chronic changes are strong predictors of renal outcomes in various kidney diseases, including IgA nephropathy. The Mayo Clinic/Renal Pathology Society suggested a glomerulonephritis reporting system with a proposal for standardized grading of chronic changes. The purpose of this study was to predict renal outcomes in patients with IgA nephropathy using chronicity grading in comparison to the Oxford classification which did not include global sclerosis. A total of 4,151 patients with IgA nephropathy were enrolled from the Korean GlomeruloNephritis Study Group registry. Chronicity grading was categorized into minimal, mild, moderate, and severe according to the extent of chronic changes. The Oxford T and S scores were considered as chronic lesions. Three prediction models were constructed: the Oxford classification model (Oxford S plus T), chronicity grading model A (chronicity grading), and chronicity grading model B (chronicity grading plus Oxford S). Using these three prediction models, the primary renal outcome (end-stage renal disease) was evaluated using Cox regression analysis and prediction performance. During the median follow-up of 6.1 (2.7–9.9) years, 304 (7.3%) patients progressed to end-stage renal disease with a cumulative incidence rate of 1.02 events per 100 person-years. In a fully adjusted multivariable model, chronicity grading was independently associated with the primary renal outcome in both models A and B. Compared to the Oxford model, both models A and B showed improvements in model fit, but not in discrimination (ΔC 0.001; 95% CI, −0.010 to 0.013 and ΔC 0.002; 95% CI, −0.005 to 0.008, respectively). Model B demonstrated improvements in integrated discrimination improvement (0.01; 95% CI, 0–0.03) and continuous net reclassification improvement (0.49; 95% CI, 0.02–0.72). The severity of chronicity grading is closely related to adverse renal outcomes in patients with IgA nephropathy, and chronicity grading could provide additional information in clinical practice alongside the Oxford classification.

## Introduction

IgA nephropathy (IgAN) is the most common primary glomerulonephritis (GN) worldwide and Korea is in a high-risk area based on genome-wide association studies presented as a world map of IgAN risk (1). According to the Korean Society of Nephrology (KSN) Registry 2017, GN is the third leading cause of developing end-stage renal disease (ESRD) and IgAN is the most frequently encountered GN in Korea currently. In Korea, the incidence of IgAN increased from 2002 to 2006, owing primarily to the indications for kidney biopsy in individuals with persistent microscopic hematuria testing positive on urinalysis (2).

However, risk stratification remains a challenge in patients with IgAN, because the natural history of IgAN ranges from persistent asymptomatic microscopic hematuria to progressive kidney failure (3). Many studies stratifying the risk factors demonstrated a consistent association with renal outcome in IgAN, both clinically with hypertension, proteinuria, and kidney function at biopsy and pathologically with Oxford classification (4–6). Oxford classification includes mesangial hypercellularity (M), endocapillary hypercellularity (E), segmental glomerulosclerosis (S), tubular atrophy/interstitial fibrosis (T), and crescents (C). Oxford classification offered the first opportunity to predict renal outcomes based on histology independent of known clinical factors, such as hypertension, proteinuria, or renal function in IgAN (7, 8). The Oxford classification provides clinicians with abundant information and is accepted worldwide.

Every GN has unique pathogenesis, but studies have shown that chronic changes are strong predictors of renal outcomes in various kidney diseases, including lupus nephritis, antineutrophil cytoplasmic autoantibody-associated vasculitis, and IgAN (9). The Mayo Clinic/Renal Pathology Society suggested a GN reporting system in 2016 with a proposal for standardized grading of chronic changes in native kidney biopsy specimens in 2017, followed by a new standardized classification and reporting of GN based on primary diagnosis, pattern of injury, score/class/grade, and chronicity grading in 2019 (9–11). A simple scoring system for chronic changes has been devised. The chronicity grading comprised four categories: glomerulosclerosis of both segmental and global sclerosis (GS), interstitial fibrosis (IF), tubular atrophy (TA), and arteriosclerosis (AS). The GS, IF, and TA scores ranged from 0 to 3 (<10%, 10–25%, 26–50%, and >50%, respectively), and the AS scores ranged from 0 to 1. Four categories were combined and divided into minimal (0–1 total score), mild (2–4 total score), moderate (5–7 total score), and severe groups (8–10 total score) (9, 10).

Given that the Oxford T lesion (corresponding to the IF and TA of chronicity grading) and Oxford S lesion had prognostic value in the renal outcome and that the Oxford classification did not include global sclerosis lesions, chronicity grading in IgAN when combined with the current Oxford classification, may strengthen the risk prediction of renal outcome. Therefore, the purpose of this study was to predict renal outcomes using chronicity grading in patients with IgAN in comparison with the Oxford classification.

## Materials and methods

### Study design and study population

The Korean GlomeruloNEphritis sTudy group (KoGNET) established a multicenter retrospective cohort of 21,697 patients who underwent renal biopsy between January 1979 and October 2018 at 18 university-affiliated hospitals in Korea (Kyungpook National University Hospital, Kyung Hee University Hospital in Gangdong, Hallym University Kangdong Sacred Heart Hospital, Gangnam Severance Hospital, Korea University Guro Hospital, Korea University Anam Hospital, Eulji University Hospital, Seoul Metropolitan Government Seoul National University Boramae Medical Center, Seoul National University Bundang Hospital, Seoul National University Hospital, Seoul St. Mary's Hospital, Severance Hospital, Pusan National University Yangsan Hospital, Ewha Womans University Mokdong Hospital, National Health Insurance Service Ilsan Hospital, Chonnam National University Hospital, Jeonbuk National University Hospital, and Hallym University Sacred Heart Hospital). Among them, 7,927 patients had either a primary or secondary pathologic diagnosis of IgAN. After applying the exclusion criteria, 4,151 patients with isolated IgAN were enrolled in the study (Supplementary Figure 1 shows the detailed study selection). This study was performed in accordance with the Declaration of Helsinki and the Declaration of Istanbul. The study was approved by the Institutional Review Board of Seoul St. Mary's Hospital (KC21ZESI0169). Written informed consent was obtained from all participants.

### Data collection and definitions

Patient demographics, laboratory investigations, and all clinical parameters, including treatment, were obtained from the KoGNET registry with electronic medical records. All demographics, laboratory data, and underlying diseases at baseline were obtained from the initial diagnosis based on kidney biopsy. The estimated glomerular filtration rate (eGFR) was calculated using the CKD-EPI equation. Instead of 24-h urine protein, spot urine protein-to-creatinine ratio (uPCR) was used to evaluate the extent of proteinuria (12). The albumin and creatinine levels were measured in grams. The mean arterial pressure (MAP) was two-thirds of the diastolic blood pressure plus one-third of the systolic blood pressure. Body mass index (BMI) is the weight in kilograms divided by the height in meters squared. A blood pressure of >140/90 mmHg was considered hypertension based on the 2020 ISH global hypertension practical guidelines (13).

### Renal histopathology and exposures

Renal biopsy specimens were examined under a light microscope, immunofluorescence, and electron microscopy, according to standard procedures. For the adequacy of renal biopsy samples, reference was made to the 2016 Oxford classification of IgAN and <8 glomeruli were excluded (14). Both monoclonal and polyclonal IgAN were classified *via* immunofluorescence staining, the former to indicate the presence of IgA only and the latter to indicate any other immunoglobulin and complement staining with IgA (11). Renal biopsies were scored according to both Oxford classification and chronicity grading. The Oxford classification of M (M0, mesangial score <0.5; M1, mesangial score >0.5), S (S0, absent; S1, present), T (T0, IF or TA ≤25%; T1, 25%< IF or TA <50%; T2, IF or TA ≥50%), and C (C0, absent; C1, 0< crescents ≤25%; C2, crescents >25%) was included in this study, but E lesions were not included because of insufficient pathological data.

The main predictor of this study was chronicity grading, comprising four categories (GS, IF, TA, and AS) of light microscopy findings that were combined and divided into minimal (0–1 total score), mild (2–4 total score), moderate (5–7 total score), and severe (8–10 total score) groups: GS score ranging from 0 to 3 (<10%, 10–25%, 26–50%, and >50%, respectively), IF score from 0 to 3 (<10%, 10–25%, 26–50%, and >50%, respectively), TA score from 0 to 3 (<10%, 10–25%, 26–50%, and >50%, respectively), and AS score from 0 to 1 (9, 10). Before conducting a primary analysis of chronicity grading with renal outcomes, the relationship between each chronic lesion was evaluated by comparing the Oxford classification with chronicity grading. A comparison of scoring based on chronic changes between the Oxford classification and chronicity grading is presented in Supplementary Table 1. Secondary analyses were done within the Oxford classification models with the inclusion of global sclerosis and in patients with both Oxford S0 and T0 scores to reveal the clinical importance of global sclerosis.

### Outcomes

The primary outcome of interest was progression to ESRD, and the renal outcomes were further analyzed with a 50% reduction in eGFR. ESRD was defined as the initiation of dialysis for a prolonged period of >3 months or kidney transplantation. A 50% reduction in eGFR was calculated from the baseline value at the time of the initial biopsy. The last visit to the outpatient department or ESRD event was considered the last follow-up time.

### Statistics and analysis

The data are presented as median (interquartile range, 25% to 75%) for continuous variables owing to non-normal distribution confirmed by the Shapiro–Wilk normality test and number (percentile) for categorical variables. The chi-squared test with Fisher's exact test for categorical variables and ANOVA analysis for continuous variables were used to determine differences in baseline characteristics and chronicity grading. Correlations between pathology variables were analyzed using the Pearson test or the Spearman test. Kaplan–Meier survival analysis with the log-rank test was used to derive survival rates. The Cox proportional hazards model was used to determine the association between chronicity grading and renal outcomes. The results are expressed as hazard ratios (HR) with 95% confidence intervals (CI). *P* < 0.05 was considered statistically significant. Three prediction models, the Oxford classification model, chronicity grading model A, and chronicity grading model B, were constructed based on the core covariates (Oxford S plus T score, chronicity grading, and chronicity grading plus Oxford S score, respectively) with sequential adjustment. To reflect the presence of segmental sclerosis, Oxford S score was included in an additional chronicity grading model (chronicity grading model B). Model 1 was adjusted for core covariates with eGFR, MAP, and uPCR, which were the strongest clinical predictors of renal outcome (4, 5, 15, 16). Model 2 (fully adjusted model) was constructed by including the same covariates as in Model 1 plus Oxford M and C scores and additionally selected for retention using a backward elimination procedure and a conservative *P* value of 0.2 (17): age, sex, smoking, diabetes mellitus (DM), BMI, and interaction between age and eGFR and between uPCR and each of MAP, sex, Oxford M/S/T/C score, and chronicity grading, and between chronicity grading and Oxford M/S/T/C score. Subsequently, to identify the impact of global sclerosis and arteriosclerosis on renal outcome, which was not included in the Oxford classification, the Oxford classification model was further constructed with the addition of global sclerosis (model 3A) or arteriosclerosis (model 3B), and the Oxford classification models were analyzed. In the secondary analysis of patients with Oxford S0 and T0, model 3 was additionally constructed with fewer variables than model 2 owing to violation of the rule of thumb. The proportional hazard assumptions were confirmed using Schoenfeld residuals. Prediction model performance was assessed using measures of model fit (Hosmer–Lemeshow χ^2^ goodness-of-fit test), calibration plots, discrimination (C statistic adapted for censoring, receiver operating characteristic [ROC] curves), and reclassification (continuous net reclassification improvement [cNRI] and integrated discrimination improvement [IDI] adapted for censoring). Calibration is the ability to correctly estimate the risk of a future event and was assessed using the Hosmer–Lemeshow χ^2^ goodness-of-fit test and *P* value of <0.05, suggesting poor model fit. Time-specific calibration was assessed by plotting the predicted vs. the observed 5-year risk of the primary renal outcome. Discrimination is the ability to differentiate between those who do and do not experience an outcome event, and was assessed using the C statistic adapted for censoring and evaluated using the 5-year risk of the primary renal outcome (approximately corresponding to the median follow-up of 6.1 years in our cohort). Reclassification was assessed using the cNRI, which evaluates the ability of a model to increase or decrease the predicted risk in those who do or do not experience an outcome event, respectively, and the IDI, which is the average improvement in sensitivity penalized for loss of specificity across all possible cut-offs. Both cNRI and IDI were adapted to account for censoring and evaluated using the 5-year risk of renal outcome. CI was generated using 100 bootstrap samples. A cNRI, IDI, or change in the C-statistic with a 95% CI that does not contain 0 can be considered statistically significant. The effect modification of chronicity grading was additionally progressed for the renal outcome with chronicity grading model B in pre-specified subgroups: uPCR at the time of biopsy (<1.0 and ≥1.0 g/g), eGFR at the time of biopsy (≥60 and <60 ml/min per 1.73 m^2^), and presence of DM. SPSS software (version 23.0; IBM Corp.) and R software (version 4.1.2; R Foundation for Statistical Computing) were used for statistical analyses. All missing data were censored on the last follow-up date.

## Results

### Baseline characteristics

Among 4,151 IgAN patients, 2,465 (59.4%) represented the minimal group under the chronicity grading, 1,201 (28.9%) were mild, 285 (6.9%) were moderate, and 200 (4.8%) were severe. Patients were followed up for a median of 6.1 (2.7–9.9) years. The median ages of the minimal, mild, moderate, and severe chronicity groups were 33 (22–45), 41 (33–51), 43 (34–52), and 42 (33–53) years, respectively; the minimal chronicity group was significantly younger than the other chronicity groups (*P* < 0.001). The severity of chronicity was correlated with a history of smoking, DM, and hypertension. Serum uric acid, total cholesterol, C4, and proteinuria tended to increase with the severity of chronicity. Conversely, renal function tended to decrease. Baseline characteristics compared with each chronicity grading are presented in Table 1 (distribution of each chronicity component according to chronicity grading is reported in Supplementary Table 2). During the follow-up, 304 (7.3%) patients progressed to ESRD with a median time of 5.1 (1.9–8.8) years, and increased severity of chronicity was linked to rapid progression to ESRD (Table 2).

**Table 1:** Baseline characteristics of 4,151 patients with IgA nephropathy, categorized by chronicity grading^a^.

	**Chronicity grading^b^ - no. (%)**					
**Characteristics**	**Minimal 2,465 (59.4)**	**Mild 1,201 (28.9)**	**Moderate 285 (6.9)**	**Severe 200 (4.8)**	**Total 4,151 (100)**	***P* value**
**Clinical characteristics at biopsy**						
Age - yr	33 (22–45)	41 (33–51)	43 (34–52)	42 (33–53)	37 (26–47)	<0.001
Male	1,303 (52.9)	587 (48.9)	139 (48.8)	108 (54.0)	2,137 (51.5)	0.09
BMI (*n* = 3,140)	23.2 (21.0–25.9)	23.7 (21.4–26.3)	23.1 (21.4–25.7)	23.5 (21.8–25.5)	23.3 (21.1–26.0)	0.018
Former/current smoker (*n* = 3,381)	404 (19.2)	219 (22.7)	50 (24.2)	44 (41.9)	717 (21.2)	<0.001
DM (*n* = 4,110)	126 (5.2)	70 (5.9)	26 (9.2)	27 (13.6)	249 (6.1)	<0.001
Hypertension (*n* = 4,119)	666 (27.3)	611 (51.2)	175 (61.6)	134 (67.0)	1,586 (38.5)	<0.001
SBP - mmHg (*n* = 3,643)	120 (110–132)	124 (113–138)	130 (120–140)	126 (117–141)	122 (112–135)	<0.001
DBP - mmHg (*n* = 3,648)	77 (70–82)	80 (70–85)	80 (72–90)	80 (70–89)	79 (70–83)	<0.001
MAP - mmHg (*n* = 3,641)	92 (83–100)	93 (85–102)	97 (88–104)	93 (86–105)	93 (83–100)	<0.001
Serum uric acid - mg/dL (*n* = 3,150)	5.6 (4.5–6.7)	6.3 (5.1–7.5)	6.9 (5.7–8.3)	7.3 (6.0–8.4)	5.9 (4.7–7.0)	<0.001
Serum total cholesterol - mg/dL (*n* = 3,724)	178 (154–209)	188 (162–218)	189 (164.5–223)	194 (162–227)	183 (157–213)	<0.001
Serum IgA - mg/dL (*n* = 3,449)	305 (241–383)	320 (261–407.5)	307 (247–406)	330 (242–424)	311 (247–395)	<0.001
Serum C3 - mg/dL (*n* = 3,650)	106 (92–121)	106 (93–121)	104 (91–119)	101 (86–117)	106 (92–121)	0.033
Serum C4 - mg/dL (*n* = 3,625)	25 (21–32)	28 (23–34)	28 (24–35)	30 (24–37)	27 (21–33)	<0.001
CKD stage						<0.001
Stage 1	1,683 (68.3)	370 (30.8)	27 (9.5)	14 (7.0)	2,094 (50.4)	
Stage 2	569 (23.1)	451 (37.6)	83 (29.1)	38 (19.0)	1,141 (27.5)	
Stage 3	159 (6.5)	318 (26.5)	124 (43.5)	67 (33.5)	668 (16.1)	
Stage 4	35 (1.4)	52 (4.3)	40 (14.0)	52 (26.0)	179 (4.3)	
Stage 5	19 (0.8)	10 (0.8)	11 (3.9)	29 (14.5)	69 (1.7)	
Serum creatinine - mg/dL	0.9 (0.7–1.0)	1.1 (0.8–1.4)	1.4 (1.1–1.9)	1.9 (1.3–2.8)	0.9 (0.8–1.2)	<0.001
eGFR - ml/min per 1.73 m^2^	104 (84–120)	74 (53–96)	52 (34–72)	37 (23–61)	90 (64–112)	<0.001
Spot uPCR - g/g (*n* = 3,468)	0.7 (0.3–1.4)	1.2 (0.7–2.3)	1.9 (1.0–3.2)	2.1 (0.9~3.9)	1.1 (0.5~2.3)	<0.001
uPCR <0.5	799 (40.3)	175 (16.5)	29 (11.6)	19 (10.7)	1,022 (29.5)	<0.001
0.5 ≤ uPCR <1.0	460 (23.2)	262 (24.7)	37 (14.9)	30 (16.9)	789 (22.8)	
1.0 ≤ uPCR <3.5	576 (29.1)	487 (45.9)	126 (50.6)	75 (42.4)	1264 (36.4)	
3.5 ≤ uPCR	147 (7.4)	136 (12.8)	57 (22.9)	53 (29.9)	393 (11.3)	
Polyclonal IgA	1,723 (69.9)	886 (73.8)	206 (72.3)	127 (63.5)	2,942 (70.9)	0.009
**Biopsy findings^c^**						
Oxford classification M1 score	1,493 (60.6)	956 (79.6)	238 (83.5)	149 (74.5)	2,836 (68.3)	<0.001
Oxford classification S1 score	352 (14.3)	569 (47.4)	152 (53.3)	91 (45.5)	1,164 (28.0)	<0.001
Oxford classification T score						<0.001
T0	2,465 (100)	1,184 (98.6)	127 (44.6)	0 (0)	3,776 (91.0)	
T1	0 (0)	5 (0.4)	53 (18.6)	2 (1.0)	60 (1.4)	
T2	0 (0)	12 (1.0)	105 (36.8)	198 (99.0)	315 (7.6)	
Oxford classification C score						<0.001
C0	2,024 (82.1)	907 (75.5)	208 (73.0)	155 (77.5)	3,553 (79.2)	
C1	416 (16.9)	260 (21.6)	67 (23.5)	40 (20.0)	849 (18.9)	
C2	25 (1.0)	34 (2.8)	10 (3.5)	5 (2.5)	81 (1.8)	

a Values for continuous variables are presented as median (interquartile range); values for categorical variables are presented as numbers (%). Missing results were excluded and the characteristics of the remaining patients are listed.

b Chronicity grading was derived from the new standardized chronicity grading system of GN based on the chronicity score: minimal (0–1), mild (2–4), moderate (5–7), and severe (8–10).

c Endocapillary lesion was not evaluated in this study.

BMI, body mass index; DM, diabetes mellitus; SBP, systolic blood pressure; DBP, diastolic blood pressure; MAP, mean arterial pressure; CKD, chronic kidney disease; eGFR, estimated glomerular filtration rate; uPCR, urine protein-to-creatinine ratio; ESRD, end-stage renal disease; GN, glomerulonephritis.

**Table 2:** Adverse outcome event rates among groups categorized by chronicity grading.

	**Total**	**Chronicity grading**			
		**Minimal**	**Mild**	**Moderate**	**Severe**
**Progression to ESRD**					
No. of patients	4,151	2,465	1,201	285	200
Person-year	29,925.3	18,336.7	8,063.1	2,064.7	1,460.8
Events (%)	304 (7.3)	50 (2.0)	112 (9.3)	67 (23.5)	75 (37.5)
Events per 100 person-yr	1.02	0.27	1.39	3.25	5.13
Median period for events (IQR)	5.1 (1.9–8.8)	7.1 (3.0–11.0)	6.0 (2.5–9.0)	4.0 (1.5–6.7)	3.0 (1.0–6.7)
**50% reduction in eGFR**					
No. of patients	3,612	2,098	1,088	259	167
Person-year	21,965.0	13,769.5	6,044.3	1,416.9	734.3
Events (%)	427 (11.8)	118 (5.6)	163 (15.0)	83 (32.0)	63 (37.7)
Events per 100 person-yr	1.94	0.86	2.70	5.86	8.58
Median period for events (IQR)	3.8 (2.0–6.5)	5.4 (2.7–7.7)	3.8 (2.0–6.3)	3.4 (1.7–5.8)	2.5 (1.3–3.8)

ESRD, end-stage renal disease; eGFR, estimated glomerular filtration rate; IQR, interquartile range.

### Relationships between each chronicity lesions

The extent of TA lesions that corresponded to each IF lesion was concordant in mild lesions (<10%: 96.7%), whereas there was some discordance in moderate-to-severe lesions (Table 3A). The relationship between global sclerosis and segmental sclerosis was discordant, and advanced global sclerosis was observed, despite the absence of segmental sclerosis (Table 3B). The association between IF and global sclerosis, which is known to have a good correlation, was concordant only in mild lesions (<10%: 93.5%) (8). In contrast, moderate and severe global sclerosis were unevenly distributed within IF lesions (Table 3C). Correlation coefficients between each chronicity lesion are shown in Table 3D. Comparisons between Oxford T score and each GS or Oxford S score and between IF/TA and GS are also presented in Table 4 and Supplementary Table 3, respectively. The majority of Oxford T0 corresponded to minimal and mild chronicity grading, and Oxford T1/T2 corresponded to moderate and severe chronicity grading, regardless of the presence of the Oxford S score (Supplementary Table 4).

**Table 3:** Comparison of interstitial fibrosis/tubular atrophy lesions and glomerulosclerosis lesions.

**A. Interstitial fibrosis and tubular atrophy**					
		**Oxford classification (T)/chronicity score (CS)**			
		**No. of patients (%)**			
		**Tubular atrophy**			
		<10%	10–25%	26–50%	>50%
Interstitial fibrosis	<10%	T0/CS0	T0/CS1	T1/CS2	T2/CS3
		3,006 (96.7)	124 (17.6)	4 (7.3)	27 (9.6)
	10–25%	T0/CS1	T0/CS2	T1/CS3	T2/CS4
		90 (2.9)	556 (78.8)	6 (10.9)	30 (7.1)
	26–50%	T1/CS2	T1/CS3	T1/CS4	T2/CS5
		1 (0.03)	8 (1.1)	41 (74.5)	2 (0.7)
	>50%	T2/CS3	T2/CS4	T2/CS5	T2/CS6
		13 (0.4)	18 (2.5)	4 (7.3)	231 (82.5)
**B. Glomerulosclerosis**						
			**Oxford classification (S)/chronicity score (CS)**			
			**No. of patients (%)**			
			**Global sclerosis**			
		0%	1–9%	10–25%	26–50%	>50%
Segmental sclerosis	0%	S0/CS0	S0/CS0	S0/CS1	S0/CS2	S0/CS3
		911 (74.5)	450 (54.6)	529 (49.0)	263 (35.4)	109 (38.5)
	1–9%	S1/CS0	S1/CS0	S1/CS1	S1/CS2	S1/CS3
		139 (11.4)	184 (22.3)	191 (17.7)	150 (20.2)	61 (21.6)
	10–25%	S1/CS1	S1/CS1	S1/CS1	S1/CS2	S1/CS3
		133 (10.9)	150 (18.2)	271 (25.1)	233 (31.4)	88 (31.1)
	26–50%	S1/CS2	S1/CS2	S1/CS2	S1/CS2	S1/CS3
		33 (2.7)	30 (3.6)	80 (7.4)	80 (10.8)	23 (8.1)
	>50%	S1/CS3	S1/CS3	S1/CS3	S1/CS3	S1/CS3
		6 (0.5)	10 (1.2)	9 (0.8)	16 (2.2)	2 (0.7)
**C. Interstitial fibrosis and global sclerosis**					
		**Global sclerosis**			
		<10%	10–25%	26–50%	>50%
Interstitial fibrosis	<10%	1912 (93.5)	809 (74.7)	359 (48.6)	81 (28.6)
	10–25%	108 (5.3)	224 (20.7)	257 (34.8)	83 (29.3)
	26–50%	3 (0.1)	9 (0.8)	23 (3.1)	17 (6.0)
	>50%	23 (1.1)	41 (3.8)	100 (13.5)	102 (36.0)
**D. Correlations between each of chronicity lesions**					
	**IF**	**TA**	**GS**	**Global sclerosis**	**Segmental sclerosis**
IF		0.86	0.47	0.49	0.20
TA			0.48	0.49	0.22
GS				0.90	0.49
Global sclerosis					0.21
Segmental sclerosis					

Correlation coefficients (R) between each chronicity lesion are shown (P < 0.001 for all).

CS, chronicity score; IF, interstitial fibrosis; TA, tubular atrophy; GS, glomerulosclerosis.

We used gray shade to differentiate T lesions (T0, T1, T2) in Table 3A, and S lesions (S0, S1) in Table 3B.

**Table 4:** Relationship between Oxford classification T score and glomerulosclerosis.

**A. Oxford classification T score and glomerulosclerosis (chronicity score)**					
		**No. of patients (%)**			
		**Glomerulosclerosis (chronicity score)**			
		**<10% (0)**	**10–25% (1)**	**26–50% (2)**	**>50% (3)**
Oxford T score	≤ 25% (T0)	1,662 (98.7)	1,214 (95.1)	721 (83.3)	179 (55.2)
	26–50% (T1)	1 (0.1)	12 (0.9)	29 (3.3)	18 (5.6)
	>50% (T2)	21 (1.2)	51 (4.0)	116 (13.4)	127 (39.2)
**B. Oxford classification T score and S score**			
		**No. of patients (%)**	
		**Segmental sclerosis (Oxford S score)**	
		**Absent (S0)**	**Present (S1)**
Oxford T score	≤ 25% (T0)	2,792 (93.5)	984 (84.5)
	26–50% (T1)	22 (0.7)	38 (3.3)
	>50% (T2)	173 (5.8)	142 (12.2)
**C. Correlations between Oxford T score and glomerulosclerosis**			
	**Glomerulosclerosis**	**global sclerosis**	**Segmental sclerosis**
Oxford T score	0.37	0.39	0.14

(A) Diverse distribution of glomerulosclerosis was observed in Oxford T0 score, in which the majority of advanced glomerulosclerosis was included. (B) The majority of segmental sclerosis was observed in Oxford T0 score. (C) Correlation coefficients (R) between Oxford T score and glomerulosclerosis are shown (P < 0.001 for all).

### Prognostic values of chronicity grading on renal outcomes

**Figure 1:**
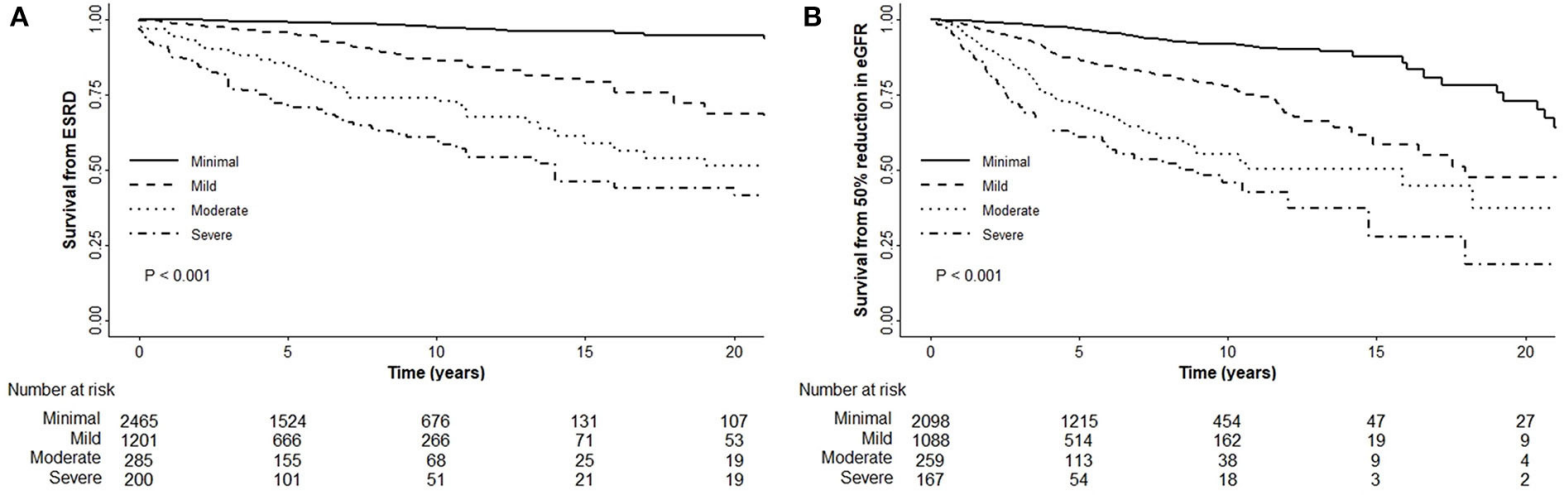
Renal outcomes with the chronicity grading. **(A)** Chronicity grading with ESRD progression. **(B)** Chronicity grading with 50% reduction in eGFR.

Kaplan–Meier analysis revealed a significant association between chronicity grading and renal outcomes, which presented as ESRD progression (*P* < 0.001) and 50% reduction in eGFR (*P* < 0.001) (Figure 1). To analyze the prognostic value of chronicity grading on renal outcome, three prediction models were first constructed, followed by Cox regression analysis with these models. The results of the univariate and multivariate Cox regression analyses are detailed in Tables 5, 6, respectively. Multivariate model 1 showed results similar to those of Model 2 in all prediction models. In the fully adjusted model 2, among the core covariates, Oxford T2 (*P* = 0.022) alone in the Oxford classification model and chronicity grading in both chronicity grading models A and B (*P* < 0.001 for all) were significantly associated with ESRD progression. Oxford C2, eGFR, and DM were significantly associated with ESRD progression in all prediction models (*P* < 0.05), and uPCR (*P* = 0.03) was significant in chronicity grading model A only.

**Table 5:** Univariate Cox regression analysis with ESRD progression.

	**Unadjusted**	
	**HR (95% CI)**	***P* value**
**eGFR**	0.96 (0.96–0.97)	<0.001
**MAP**	1.02 (1.02–1.03)	<0.001
**uPCR**	1.15 (1.12–1.19)	<0.001
**Chronicity grading**		
Minimal	1 (reference)	
Mild	5.08 (3.64–7.10)	<0.001
Moderate	12.04 (8.34–17.39)	<0.001
Severe	19.16 (13.38–27.44)	<0.001
**Oxford classification**		
S1	2.77 (2.21–3.47)	<0.001
T1	4.06 (2.46–6.71)	<0.001
T2	6.07 (4.75–7.76)	<0.001
M1	1.61 (1.24–2.08)	<0.001
C1	1.49 (1.15–1.94)	<0.001
C2	5.04 (3.15–8.06)	<0.001
**Age**	1.03 (1.02–1.04)	<0.001
**Male**	1.30 (1.04–1.64)	0.023
**DM**	3.22 (2.31–4.51)	<0.001
**Individual chronicity score**	
GS1	3.47 (2.25–5.34)	<0.001
GS2	9.16 (6.08–13.81)	<0.001
GS3	21.07 (13.90–31.92)	<0.001
IF1	4.46 (3.38–5.87)	<0.001
IF2	7.81 (4.67–13.08)	<0.001
IF3	8.82 (6.61–11.76)	<0.001
TA1	5.39 (4.07–7.14)	<0.001
TA2	7.55 (4.53–12.58)	<0.001
TA3	10.50 (7.84–14.08)	<0.001
AS1	1.98 (1.51–2.60)	<0.001
**Former/current smoker**	1.87 (1.42–2.48)	<0.001

ESRD, end-stage renal disease; HR, hazard ratio; CI, confidence interval; eGFR, estimated glomerular filtration rate; MAP, mean arterial pressure; uPCR, urine protein-to-creatinine ratio; DM, diabetes mellitus; GS, glomerulosclerosis; IF, interstitial fibrosis; TA, tubular atrophy; AS, arteriosclerosis.

**Table 6:** Association of chronicity grading and oxford classification with ESRD progression.

	**(A) Oxford classification model**				**(B) Chronicity grading model A**				**(C) Chronicity grading model B**			
	**Model 1**		**Model 2**		**Model 1**		**Model 2**		**Model 1**		**Model 2**	
	**HR (95% CI)**	***P* value**	**HR (95% CI)**	***P* value**	**HR (95% CI)**	***P* value**	**HR (95% CI)**	***P* value**	**HR (95% CI)**	***P* value**	**HR (95% CI)**	***P* value**
**No. of events/total**	229/3,027		223/2,995		229/3,027		223/2,995		229/3,027		223/2,995	
eGFR	0.97 (0.96–0.97)	<0.001	0.97 (0.96–0.97)	<0.001	0.97 (0.97–0.98)	<0.001	0.97 (0.97–0.98)	<0.001	0.97 (0.97–0.98)	<0.001	0.97 (0.97–0.98)	<0.001
MAP	1.00 (0.99–1.01)	0.658	1.00 (0.99–1.01)	0.875	1.00 (0.99–1.00)	0.328	1.00 (0.99–1.00)	0.389	1.00 (0.99–1.01)	0.494	1.00 (0.99–1.01)	0.654
uPCR	1.06 (1.00–1.12)	0.063	1.04 (0.98–1.11)	0.164	1.06 (1.02–1.11)	0.005	1.05 (1.01–1.10)	0.030	1.05 (0.98–1.11)	0.148	1.03 (0.96–1.10)	0.434
**Chronicity grading**											
Minimal					1 (reference)	<0.001	1 (reference)	<0.001	1 (reference)	<0.001	1 (reference)	<0.001
Mild					2.85 (1.90–4.27)		2.81 (1.83–4.31)		2.55 (1.69–3.87)		2.51 (1.62–3.91)	
Moderate					4.07 (2.59–6.40)		4.22 (2.61–6.81)		3.42 (2.13–5.49)		3.56 (2.16–5.86)	
Severe					4.18 (2.61–6.68)		4.39 (2.68–7.19)		3.65 (2.26–5.91)		3.85 (2.33–6.39)	
**Oxford classification**											
S1	1.62 (1.13–2.32)	0.009	1.64 (1.13–2.39)	0.009					1.28 (0.88–1.86)	0.193	1.27 (0.86–1.88)	0.222
T1	1.08 (0.61–1.91)	0.024	1.07 (0.60–1.91)	0.022								
T2	1.46 (1.05–2.01)		1.47 (1.06–2.04)									
M1			1.18 (0.85–1.64)	0.33			1.08 (0.77–1.51)	0.667			0.99 (0.71–1.39)	0.965
C1			1.02 (0.75–1.40)	0.180			1.13 (0.83–1.54)	0.031			1.07 (0.79–1.47)	0.060
C2			2.03 (1.09–3.77)				2.00 (1.08–3.73)				2.13 (1.14–3.98)	
**Age**			1.00 (0.99–1.01)	0.501			0.99 (0.98–1.00)	0.264			1.00 (0.99–1.01)	0.447
**Male**			1.30 (0.99–1.70)	0.063			1.23 (0.94–1.62)	0.128			1.30 (0.99–1.71)	0.061
**DM**			1.66 (1.05–2.62)	0.031			1.63 (1.04–2.58)	0.035			1.69 (1.07–2.66)	0.025
uPCR*Oxford S		0.314		0.261						0.322		0.195

Multivariate Cox regression analysis of (A) Oxford S and T scores, (B) chronicity grading, and (C) chronicity grading and Oxford S score with ESRD progression. Model 1 was adjusted for eGFR, MAP, uPCR, and interaction terms. Fully adjusted Model 2 contained predictors of Model 1 plus Oxford M, Oxford C score, and additional predictors (age, sex, DM, and interaction term). Additional predictors of model 2 were selected using backward elimination chosen among age, sex, smoking, DM, BMI, and interaction between age and eGFR and between uPCR and each of MAP, sex, Oxford M/S/T/C score, and chronicity grading, and between chronicity grading and Oxford M/S/T/C score.

ESRD, end-stage renal disease; HR, hazard ratio; CI, confidence interval; eGFR, estimated glomerular filtration rate; MAP, mean arterial pressure; uPCR, urine protein-to-creatinine ratio; DM, diabetes mellitus; BMI, body mass index.

### Prediction performance of the chronicity grading models

**Figure 2:**
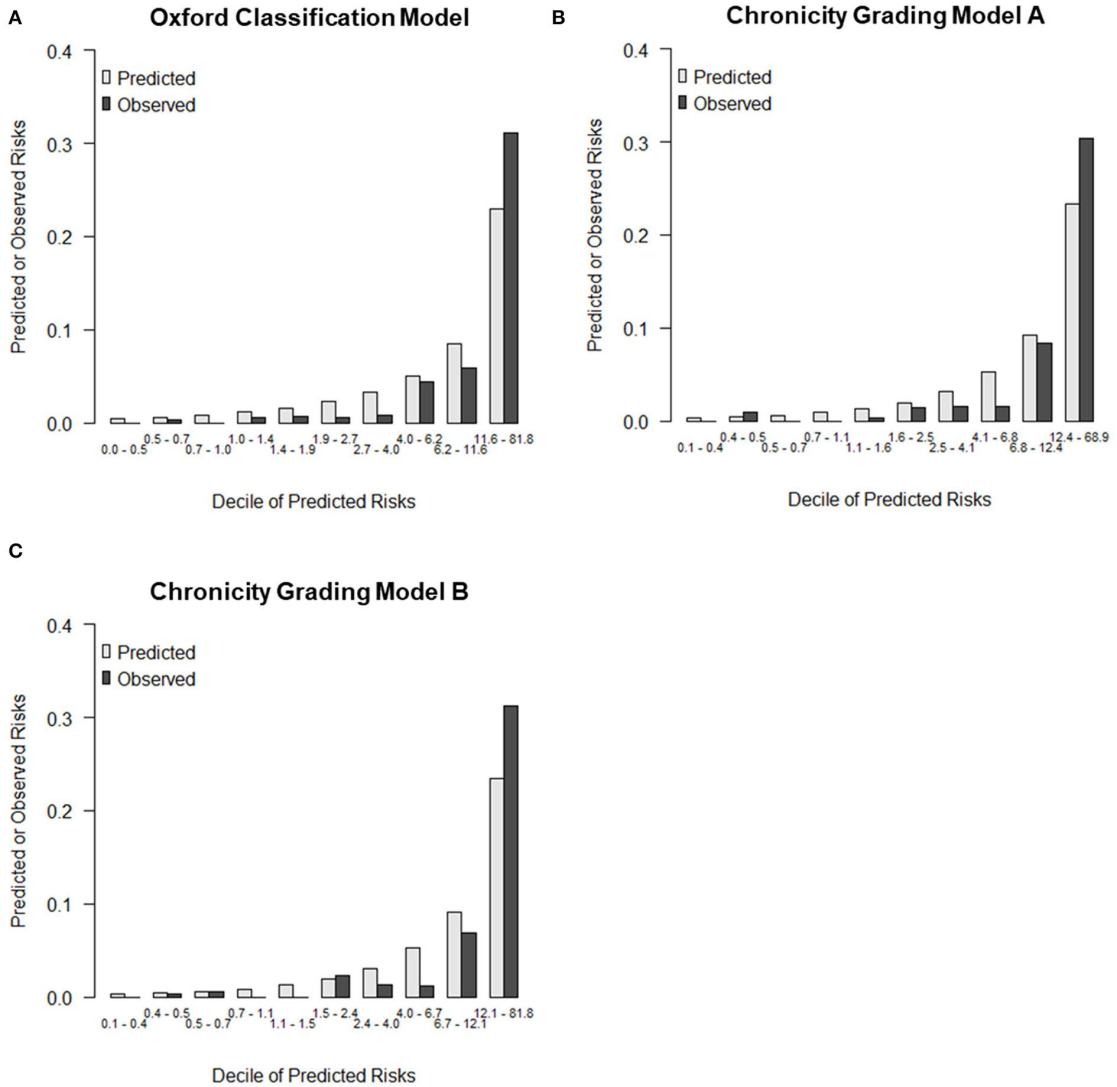
Calibration plot for predicted vs. observed 5-year risks of the primary renal outcome. **(A)** Calibration plots by deciles of predicted risk for Oxford classification model, **(B)** Chronicity grading model A, and **(C)** Chronicity grading model B.

The details of the prediction performance are presented in Table 7. Both chronicity grading models A and B qualified in model fit with the Hosmer–Lemeshow χ^2^ goodness-of-fit test (*P* = 0.135 and *P* = 0.449, respectively). The corresponding calibration plot for the predicted vs. observed 5-year risks of the primary renal outcome in each model is presented in Figure 2. Compared with the Oxford classification model, chronicity grading model A was not significant in discrimination with the C statistic and in reclassification assessed by both the IDI and cNRI. Chronicity grading model B, compared with the Oxford classification model, showed significant improvement in reclassification, as assessed by both the IDI (0.01; 95% CI, 0 to 0.03) and the cNRI (0.49; 95% CI, 0.02–0.72), but was not significant in discrimination with the C statistic (ΔC 0.002; 95% CI, −0.005–0.008). The discrimination of each model with ROC curves is presented in Supplementary Figure 2.

**Table 7:** Prediction performance of the chronicity grading models compared with the oxford classification model^a^.

**Variable**	**Oxford classification model^b^**	**Chronicity grading model^c^**	
		**Model A**	**Model B**
Hosmer-Lemeshow χ^2^ goodness-of-fit test	0.591	0.135	0.449
AIC	2,824	2,798	2,793
C statistic	0.90 (0.87–0.93)	0.90 (0.87–0.93)	0.90 (0.87–0.93)
**Model performance compared with the Oxford classification**			
ΔC statistic		0.001 (−0.010–0.013)	0.002 (−0.005–0.008)
Continuous NRI		0.03 (−0.43–0.56)	0.49 (0.02–0.72)
NRI (events)		0.02 (−0.20–0.26)	0.23 (−0.04–0.40)
NRI (non-events)		0.01 (−0.33–0.35)	0.26 (0.03–0.37)
IDI		0.01 (−0.02–0.03)	0.01 (0–0.03)

a Data are reported as measure (95% CI). Calibration was assessed using the Hosmer–Lemeshow χ^2^goodness-of-fit test, and P value of <0.05 suggesting a poor model fit. Discrimination was assessed using the C statistic; and reclassification using the IDI and overall continuous NRI. For the change(Δ) in the C statistic, continuous NRI, and IDI, a statistically significant improvement was indicated by a 95% CI that did not include 0.

b The Oxford classification model was fully adjusted for eGFR, MAP, uPCR, Oxford M/S/T/C score, age, sex, DM, and the interaction between uPCR and Oxford S score.

c Chronicity grading model A is similar to the Oxford classification model except for chronicity grading instead of Oxford S/T score; chronicity grading model B contains model A plus Oxford S score.

AIC, Akaike information criterion; NRI, net reclassification improvement; IDI, integrated discrimination improvement.

### Secondary analysis

To confirm our hypothesis that global sclerosis had a significant impact on renal outcomes regardless of IF, TA, or segmental sclerosis, a secondary analysis was performed in two ways. In the Oxford classification model analysis, global sclerosis (in model 3A) had the highest risk for ESRD, which concurrently led to insignificant results of the Oxford T score, whereas arteriosclerosis (in model 3B) was not significant (Table 8). In the analysis of a subset of 1915 patients with both Oxford S0 and T0, the severity of chronicity grading was significantly associated with ESRD progression, with the highest risk among the other factors in all chronicity grading models. Severe chronicity grading (chronicity grading score 8–10) was not observed in the analysis because the chronicity grading score of 6 was the highest score among participants (GS 3, IF 1, TA 1, and AS 1). Chronicity grading model 3 was constructed in consideration of potential bias in chronicity grading model 2; however, there were consistent results among the chronicity grading models. In both the Oxford classification model and chronicity grading model, DM was significantly associated with ESRD progression with a higher HR than that observed in the primary analysis (Table 9).

**Table 8:** Impact of global sclerosis in addition to Oxford classification on ESRD progression.

	**Oxford classification model**					
	**Model 2**		**Model 3A**		**Model 3B**	
**AIC**	2,824		2,785		2,825	
**C statistic**	0.90 (0.87–0.93)		0.90 (0.87–0.92)		0.90 (0.87–0.93)	
	**HR (95% CI)**	***P*** **value**	**HR (95% CI)**	***P*** **value**	**HR (95% CI)**	***P*** **value**
**No. of events/total**	223/2,995		223/2,995		223/2,995	
**eGFR**	0.97 (0.96–0.97)	<0.001	0.97 (0.97–0.98)	<0.001	0.97 (0.96–0.97)	<0.001
**MAP**	1.00 (0.99–1.01)	0.875	1.00 (0.99–1.01)	0.543	1.00 (0.99–1.01)	0.867
**uPCR**	1.04 (0.98–1.11)	0.164	1.03 (0.96–1.10)	0.453	1.04 (0.98–1.11)	0.158
**Chronicity grading score**						
Global sclerosis 1			1.80 (1.02–3.19)	<0.001		
Global sclerosis 2			3.54 (2.03–6.17)			
Global sclerosis 3			5.29 (2.91–9.60)			
Arteriosclerosis 1					1.06 (0.76–1.47)	0.735
**Oxford classification**						
S1	1.64 (1.13–2.39)	0.009	1.21 (0.81–1.80)	0.346	1.65 (1.13–2.39)	0.009
T1	1.07 (0.60–1.91)	0.022	0.82 (0.45–1.48)	0.922	1.06 (0.59–1.90)	0.024
T2	1.47 (1.06–2.04)		1.01 (0.71–1.44)		1.46 (1.05–2.04)	
M1	1.18 (0.85–1.64)	0.330	1.06 (0.75–1.48)	0.751	1.18 (0.85–1.64)	0.333
C1	1.02 (0.75–1.40)	0.180	1.14 (0.84–1.57)	0.030	1.02 (0.75–1.40)	0.180
C2	2.03 (1.09–3.77)		2.50 (1.34–4.67)		2.02 (1.09–3.77)	
**Age**	1.00 (0.99–1.01)	0.501	1.00 (0.99–1.01)	0.494	1.00 (0.99–1.01)	0.472
**Male**	1.30 (0.99–1.70)	0.063	1.32 (1.01–1.74)	0.045	1.29 (0.98–1.70)	0.074
**DM**	1.66 (1.05–2.62)	0.031	1.81 (1.14–2.86)	0.011	1.64 (1.03–2.60)	0.036
uPCR*Oxford S		0.261		0.179		0.286

Additional model adjustment for the previous Oxford classification model 2 was as follows: Model 3A was adjusted for model 2 plus the chronicity grading of global sclerosis. Model 3B was adjusted for model 2 plus the chronicity grading of arteriosclerosis.

ESRD, end-stage renal disease; AIC, Akaike information criterion; HR, hazard ratio; CI, confidence interval; eGFR, estimated glomerular filtration rate; MAP, mean arterial pressure; uPCR, urine protein-to-creatinine ratio; DM, diabetes mellitus.

**Table 9:** Association of chronicity grading and Oxford classification with ESRD progression in patients with both Oxford S0 and T0.

	**Oxford classification model**				**Chronicity grading model**					
	**Model 1**		**Model 2**		**Model 1**		**Model 2**		**Model 3^b^**	
	**HR (95% CI)**	***P* value**	**HR (95% CI)**	***P* value**	**HR (95% CI)**	***P* value**	**HR (95% CI)**	***P* value**	**HR (95% CI)**	***P* value**
**No. of events/total**	72/1,915		69/1,894		72/1,915		69/1,894		69/1,894	
**eGFR**	0.97 (0.96–0.97)	<0.001	0.97 (0.96–0.97)	<0.001	0.97 (0.96–0.98)	<0.001	0.97 (0.96–0.98)	<0.001	0.97 (0.96–0.98)	<0.001
**MAP**	1.00 (0.98–1.02)	0.972	1.00 (0.98–1.02)	0.874	1.00 (0.98–1.02)	0.938	1.00 (0.99–1.02)	0.662	1.00 (0.99–1.02)	0.645
**uPCR**	1.07 (1.00–1.14)	0.043	1.05 (0.98–1.13)	0.164	1.07 (0.99–1.15)	0.083	1.05 (0.97–1.14)	0.267	1.04 (0.96–1.13)	0.308
**Chronicity grading**										
Minimal					1 (reference)	<0.001	1 (reference)	<0.001	1 (reference)	<0.001
Mild					2.69 (1.59–4.56)		2.73 (1.55–4.82)		2.59 (1.51–4.46)	
Moderate					9.76 (4.81–19.79)		11.59 (5.48–24.49)		11.00 (5.32–22.72)	
Severe					N/A^a^		N/A^a^		N/A^a^	
**Oxford classification**										
M1			1.30 (0.76–2.22)	0.338			0.95 (0.55–1.66)	0.860		
C1			1.11 (0.62–1.99)	0.602			1.14 (0.64–2.05)	0.402	1.12 (0.63–2.01)	0.463
C2			1.31 (0.42–4.12)				1.75 (0.55–5.62)		1.67 (0.52–5.37)	
**Age**			1.00 (0.98–1.02)	0.865			0.99 (0.97–1.01)	0.450		
**Male**			1.01 (0.62–1.65)	0.976			1.08 (0.66–1.75)	0.759		
**DM**			2.47 (1.21–5.06)	0.013			3.16 (1.57–6.37)	0.001	3.00 (1.52–5.92)	0.002

Multivariate Cox regression analysis of patients with S0 and T0. Model 1 was adjusted for eGFR, MAP, and uPCR. Fully adjusted Model 2 contained predictors of Model 1 plus Oxford M, Oxford C score, and additional predictors (age, sex, and DM). Additional predictors of model 2 were selected using backward elimination chosen among age, sex, smoking, DM, BMI, and interaction between age and eGFR and between uPCR and each of MAP, sex, Oxford M/C score, and chronicity grading, and between chronicity grading and Oxford M/C score.

a No patients were observed in the severe chronicity grading; because the chronicity grading score of 6 was the highest score among participants.

b Model 3 was modified with six variables from Model 2, excluding Oxford M score, age, and sex which were least significant, owing to violation of the rule of thumb.

ESRD, end-stage renal disease; HR, hazard ratio; CI, confidence interval; eGFR, estimated glomerular filtration rate; MAP, mean arterial pressure; uPCR, urine protein-to-creatinine ratio; DM, diabetes mellitus; BMI, body mass index.

### Subgroup analysis

**Figure 3:**
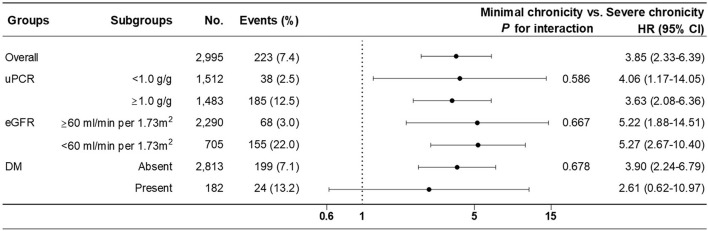
Subgroup analysis of renal outcome (progression to ESRD) between minimal (reference) and severe chronicity grading from fully adjusted chronicity grading model B.

Subgroup analysis of ESRD progression was performed between the minimal chronicity group and the severe chronicity group of chronicity grading model B in the pre-specified subgroups. There were no significant interactions among subgroups stratified by uPCR (*P* = 0.586), eGFR (*P* = 0.667), or DM (*P* = 0.678) (Figure 3).

## Discussion

IgAN was analyzed based on renal outcomes under chronicity grading, which was intended to standardize the chronic changes in GN. Our study can be summarized as follows: first, the relationships between each chronic lesion were evaluated. A poor correlation between IF/TA lesions and glomerulosclerosis lesions, including the correlation between IF and global sclerosis was confirmed. Second, the chronicity grading was significantly associated with ESRD progression. Third, compared with the Oxford classification model, the chronicity grading model, which included chronicity grading instead of the Oxford T score, showed significant improvement in reclassification despite statistically insignificant discrimination.

The Oxford classification system has been the mainstay of histological evaluation in patients with IgAN since 2009. This classification was originally proposed based on various factors correlated with each of the pathological features, reproducibility, association with renal outcomes from a cohort study, correlations between pathologic lesions and clinical presentation/outcome, interaction between pathologic features and therapy, and clinical factors (7). Such profound analysis prompted clinicians to implement Oxford classification in clinical practice currently, resulting in the publication of many validation studies demonstrating that T lesions were consistent and independent predictors of renal outcomes, whereas S lesions had variable results for renal outcomes. In our study, Oxford T2 was significantly associated with renal outcome, but T1 was not, probably because of the relatively small number of T1 patients (60 out of 4151). The Oxford S score was not significant in our study and was consistent with that in our previously published retrospective study (18).

Grading chronicity is an extremely important step, and chronic changes are incorporated into many GN classification systems (10). The major components of chronic changes include GS, IF, and TA. Several differences in chronicity existed in the GN reporting system compared with the current Oxford classification because the Oxford classification is a dynamic score and is not intended to separate acute and chronic lesions. Both Oxford T and S lesions represent chronic changes, although tip lesions and podocyte hypertrophy of S lesions are acute active lesions. For GS lesions, the Oxford classification only included segmental glomerulosclerosis, whereas the chronicity grading incorporated global glomerulosclerosis and was subdivided into four classes, based on the extent of sclerosis. Oxford T lesions were defined as either IF or TA, whichever was greater, whereas the chronicity grading proposed distinct IF and TA lesions, with a score below 10% suggesting absence. AS lesions that were not included in the Oxford classification were added to the chronicity grading.

In the process of defining the Oxford classification in 2009, 23 pathology variables had significant correlations, and among them, two closely linked variables were focused upon: IF and TA (*R* = 0.98) and IF and global glomerulosclerosis (*R* = 0.80) (8). In our study, 3,834 (92.1%) patients had a good correlation between IF and TA, but only 2,261 (54.5%) patients had a good correlation between IF and global sclerosis (Table 3). Although IF/TA was preferred to global sclerosis as their quantification is less susceptible to error in Oxford classification, neither interstitial fibrosis nor Oxford T scores reflected glomerulosclerosis in our study (Supplementary Tables 3, 4) (8). In our study, the relationship between global sclerosis and segmental sclerosis was not consistent with the extent of the lesions (Table 3). Furthermore, a correlation between poor renal outcome and both the extent of GS lesions and each component of GS lesions (segmental sclerosis, global sclerosis, and combined segmental and global sclerosis) was established (Supplementary Figure 3). These results indicate the need for reevaluating glomerulosclerosis lesions, including global sclerosis. In our study, the significant impact of global sclerosis on renal outcomes within the Oxford classification models was also identified (Table 8). Consequently, it was found that the severity of chronicity grading was associated with poor renal outcomes in patients with both Oxford T0 and S0 (Table 9), in which global sclerosis was the major pathology of chronicity grading.

By applying chronicity grading to our analysis, a significant association between chronicity grading and ESRD progression was demonstrated. However, the chronicity grading was not in the original Oxford classification, which is well established and has numerous qualified validations. To verify the prediction performance of our chronicity grading models, a reference was made to the Oxford classification model from the new international IgAN risk-prediction tool (19). Since our study emphasized chronic changes, a model was constructed with a core covariate containing Oxford T and S scores in the Oxford classification model; furthermore, two chronicity grading models were constructed based on whether segmental sclerosis was present or not. Interestingly, the model that contained chronicity grading only as a core covariate (chronicity grading model A) was not significant in overall prediction performance, but the model that contained chronicity grading and Oxford S score as core covariates (chronicity grading model B) showed a significant improvement in reclassification.

Although worse renal function might be associated with chronic changes and further progression to ESRD, chronic changes still exist in patients with preserved renal function. The number of patients with eGFR >60 with minimal, mild, moderate, and severe chronicity was 2,252 (69.6%), 821 (25.4%), 110 (3.4%), and 52 (1.6%), respectively, indicating that approximately 30% of patients with eGFR >60 had chronic changes, at least to a certain extent. Therefore, a subgroup analysis was performed to identify interactions; no significant interactions were found among subgroups stratified by uPCR, eGFR, and DM.

Our study had several limitations. First, owing to insufficient data in our cohort, parameters of the Oxford E score, use of renin-angiotensin system blockade, and use of immunosuppression were excluded, which are known to have a consistent association with renal outcomes and are basic components of the international IgAN risk-prediction model. Second, because of the nature of this retrospective cohort study, it is possible that potential confounders were not entirely controlled initially. Third, our cohort included only a single measurement of blood pressure at the time of biopsy, which might have affected the insignificant results. In conclusion, the severity of chronicity grading is closely related to adverse renal outcomes in patients with IgAN, and chronicity grading can provide additive information along with the Oxford classification in clinical practice.

## Data availability statement

The original contributions presented in the study are included in the article/Supplementary material, further inquiries can be directed to the corresponding author.

## Ethics statement

The studies involving human participants were reviewed and approved by Institutional Review Board of Seoul St. Mary's Hospital (KC21ZESI0169). The patients/participants provided their written informed consent to participate in this study.

## Author contributions

DK wrote the manuscript and analyzed the data. BC revised the manuscript and conceived and designed the study. DK, TB, HC, HL, SO, CP, and CY collected the data. All authors reviewed the manuscript. All authors contributed to the article and approved the submitted version.

## Funding

This research was supported by the Basic Science Research Program through the National Research Foundation of Korea (NRF), funded by the Ministry of Science, ICT & Future Planning (NRF-2020R1F1A1049130). This work was supported by Cooperative Research Grant 2017 from the Korean Society of Nephrology. The sponsor had no role in the study design, data collection and analysis, decision to publish, or manuscript preparation.

## Conflict of interest

The authors declare that the research was conducted in the absence of any commercial or financial relationships that could be construed as a potential conflict of interest.

## Publisher's note

All claims expressed in this article are solely those of the authors and do not necessarily represent those of their affiliated organizations, or those of the publisher, the editors and the reviewers. Any product that may be evaluated in this article, or claim that may be made by its manufacturer, is not guaranteed or endorsed by the publisher.
