# Supplementary material for: Prognostic value of chronicity grading on renal outcomes in patients with IgA nephropathy
Source: Front Med (Lausanne). 2022 Aug 24;9:952050. doi: 10.3389/fmed.2022.952050 (PMC9449317; doi:10.3389/fmed.2022.952050)
Supplement: Supplementary file 1 [file Data_Sheet_1.docx]

**Supplementary Materials**

**Supplementary Table 1.** Comparison of scoring based on chronic changes between Oxford classification and chronicity grading

**Supplementary Table 2.** Distribution of chronic changes categorized by chronicity grading

**Supplementary Table 3.** Relationship between glomerulosclerosis and interstitial fibrosis/tubular atrophy in the chronicity grading system

**Supplementary Table 4.** Distribution of Oxford classification according to chronicity score

**Supplementary Figure 1.** Study selection

**Supplementary Figure 2.** ROC curves for predicting ESRD progression at 5 years

**Supplementary Figure 3.** Renal outcome with glomerulosclerosis lesions

**Supplementary Table 1. Comparison of scoring based on chronic changes between Oxford classification and chronicity grading**

| **Chronic Changes** |  | **Oxford classification** | **Chronicity grading** |
| --- | --- | --- | --- |
| **Global sclerosis** | 0% | S0 or S1 | GS0 or GS1 or GS2 or GS3 |
|  | 1-9% | S0 or S1 | GS0 or GS1 or GS2 or GS3 |
|  | 10-25% | S0 or S1 | GS1 or GS2 or GS3 |
|  | 26%-50% | S0 or S1 | GS2 or GS3 |
|  | >50% | S0 or S1 | GS3 |
| **Segmental sclerosis** | 0% | S0 | GS0 or GS1 or GS2 or GS3 |
|  | 1-9% | S1 | GS0 or GS1 or GS2 or GS3 |
|  | 10-25% | S1 | GS1 or GS2 or GS3 |
|  | 26%-50% | S1 | GS2 or GS3 |
|  | >50% | S1 | GS3 |
| **Interstitial fibrosis** | <10% | T0 or T1 or T2 | IF0 |
|  | 10-25% | T0 or T1 or T2 | IF1 |
|  | 26%-50% | T1 or T2 | IF2 |
|  | >50% | T2 | IF3 |
| **Tubular atrophy** | <10% | T0 or T1 or T2 | TA0 |
|  | 10-25% | T0 or T1 or T2 | TA1 |
|  | 26%-50% | T1 or T2 | TA2 |
|  | >50% | T2 | TA3 |
| **Arteriosclerosis** |  |  |  |
| Intimal thickening < thickness of media | | N/A | AS0 |
| Intimal thickening ≥ thickness of media | | N/A | AS1 |

Abbreviations: GS, glomerulosclerosis; IF, interstitial fibrosis; TA, tubular atrophy; AS, arteriosclerosis; N/A, not applicable.

**Supplementary Table 2. Distribution of chronic changes categorized by chronicity grading**

|  |  | **The chronicity grading** | | | | | ***P* value** |
| --- | --- | --- | --- | --- | --- | --- | --- |
| **The chronicity Score** | | **Minimal** | **Mild** | **Moderate** | **Severe** | **Total** |  |
| Glomerulosclerosis | 0 | 1610 (65.3) | 58 (4.8) | 16 (5.6) | 0 (0) | 1684 (40.6) | < 0.001 |
|  | 1 | 855 (34.7) | 370 (30.8) | 43 (15.1) | 9 (4.5) | 1277 (30.8) |  |
|  | 2 | 0 (0) | 671 (55.9) | 105 (36.8) | 90 (45.0) | 866 (20.9) |  |
|  | 3 | 0 (0) | 102 (8.5) | 121 (42.5) | 101 (50.5) | 324 (7.8) |  |
| Interstitial fibrosis | 0 | 2459 (99.8) | 673 (56.0) | 29 (10.2) | 0 (0) | 3161 (76.2) | < 0.001 |
|  | 1 | 6 (0.2) | 520 (43.3) | 146 (51.2) | 0 (0) | 672 (16.2) |  |
|  | 2 | 0 (0) | 3 (0.2) | 45 (15.8) | 4 (2.0) | 52 (1.3) |  |
|  | 3 | 0 (0) | 5 (0.4) | 65 (22.8) | 196 (98.0) | 266 (6.4) |  |
| Tubular atrophy | 0 | 2453 (99.5) | 643 (53.5) | 14 (4.9) | 0 (0) | 3110 (74.9) | < 0.001 |
|  | 1 | 12 (0.5) | 548 (45.6) | 146 (51.2) | 0 (0) | 705 (17.0) |  |
|  | 2 | 0 (0) | 3 (0.2) | 48 (16.8) | 4 (2.0) | 55 (1.3) |  |
|  | 3 | 0 (0) | 7 (0.6) | 77 (27.0) | 196 (98.0) | 280 (6.7) |  |
| Arteriosclerosis | 0 | 2384 (96.7) | 946 (78.8) | 174 (61.1) | 131 (65.5) | 3635 (87.6) | < 0.001 |
|  | 1 | 81 (3.3) | 255 (21.2) | 111 (38.9) | 69 (34.5) | 516 (12.4) |  |

**Supplementary Table 3. Relationship between glomerulosclerosis and interstitial fibrosis/tubular atrophy in the chronicity grading system**

|  |  | chronicity score No. of patients (%) | | | |
| --- | --- | --- | --- | --- | --- |
|  |  | **Glomerulosclerosis (chronicity score)** | | | |
|  |  | <10% (0) | 10-25% (1) | 26%-50% (2) | >50% (3) |
| **Interstitial  fibrosis (chronicity score)** | <10% (0) | 0 | 1 | 2 | 3 |
|  |  | 1611 (95.7) | 1002 (78.5) | 443 (51.2) | 105 (34.2) |
|  | 10-25% (1) | 1 | 2 | 3 | 4 |
|  |  | 53 (3.1) | 229 (17.9) | 296 (34.2) | 94 (29.0) |
|  | 26%-50% (2) | 2 | 3 | 4 | 5 |
|  |  | 1 (0.1) | 8 (0.6) | 26 (3.0) | 17 (5.2) |
|  | >50% (3) | 3 | 4 | 5 | 6 |
|  |  | 19 (1.1) | 38 (3.0) | 101 (11.7) | 108 (33.3) |
| **Tubular atrophy (chronicity score)** | <10% (0) | 0 | 1 | 2 | 3 |
|  |  | 1601 (95.7) | 987 (77.3) | 424 (49.0) | 98 (30.2) |
|  | 10-25% (1) | 1 | 2 | 3 | 4 |
|  |  | 64 (3.8) | 236 (18.5) | 311 (35.9) | 95 (29.3) |
|  | 26%-50% (2) | 2 | 3 | 4 | 5 |
|  |  | 3 (0.2) | 9 (0.7) | 27 (3.1) | 16 (4.9) |
|  | >50% (3) | 3 | 4 | 5 | 6 |
|  |  | 16 (1.0) | 45 (3.5) | 104 (12.0) | 115 (35.5) |
| **Chronicity score of combined IF/TA** | 0 | 0 | 1 | 2 | 3 |
|  |  | 1592 (94.5) | 960 (75.2) | 379 (43.8) | 75 (23.1) |
|  | 1 | 1 | 2 | 3 | 4 |
|  |  | 24 (1.4) | 57 (4.5) | 95 (11.0) | 38 (11.7) |
|  | 2 | 2 | 3 | 4 | 5 |
|  |  | 46 (2.7) | 199 (15.6) | 248 (28.6) | 68 (21.0) |
|  | 3 | 3 | 4 | 5 | 6 |
|  |  | 4 (0.2) | 15 (1.2) | 18 (2.1) | 17 (5.2) |
|  | 4 | 4 | 5 | 6 | 7 |
|  |  | 2 (0.1) | 14 (1.1) | 36 (4.2) | 27 (8.3) |
|  | 5 | 5 | 6 | 7 | 8 |
|  |  | 2 (0.1) | 0 (0) | 1 (0.1) | 3 (0.9) |
|  | 6 | 6 | 7 | 8 | 9 |
|  |  | 14 (0.8) | 32 (2.5) | 89 (10.3) | 96 (29.6) |

Abbreviation: IF/TA, interstitial fibrosis and tubular atrophy.

**Supplementary Table 4. Distribution of Oxford classification according to chronicity score^a^**

|  | | No. of patients (%) | | | | | |
| --- | --- | --- | --- | --- | --- | --- | --- |
|  |  | **Oxford classification** | | | | | |
| **Chronicity score (chronicity grade)** | | T0 | | T1 | | T2 | |
|  |  | S0 | S1 | S0 | S1 | S0 | S1 |
| 0 | Minimal | 1511 (54.1) | 0 (0) | 0 (0) | 0 (0) | 0 (0) | 0 (0) |
| 1 |  | 602 (21.6) | 352 (35.8) | 0 (0) | 0 (0) | 0 (0) | 0 (0) |
| 2 | Mild | 311 (11.1) | 211 (21.4) | 0 (0) | 0 (0) | 0 (0) | 0 (0) |
| 3 |  | 177 (6.3) | 188 (19.1) | 2 (9.1) | 0 (0) | 4 (2.3) | 0 (0) |
| 4 |  | 131 (4.7) | 166 (16.9) | 2 (9.1) | 1 (2.6) | 5 (2.9) | 3 (2.1) |
| 5 | Moderate | 52 (1.9) | 59 (6.0) | 4 (18.2) | 10 (26.3) | 13 (7.5) | 9 (6.3) |
| 6 |  | 8 (0.3) | 8 (0.8) | 4 (18.2) | 13 (34.2) | 19 (11.0) | 18 (12.7) |
| 7 |  | 0 (0) | 0 (0) | 8 (36.4) | 14 (36.8) | 25 (14.5) | 21 (14.8) |
| 8 | Severe | 0 (0) | 0 (0) | 2 (9.1) | 0 (0) | 39 (22.5) | 37 (26.1) |
| 9 |  | 0 (0) | 0 (0) | 0 (0) | 0 (0) | 51 (29.5) | 40 (28.2) |
| 10 |  | 0 (0) | 0 (0) | 0 (0) | 0 (0) | 17 (9.8) | 14 (9.9) |

^a^Chronicity grading of arteriosclerosis was not included in the table.

**Supplementary Figure 1. Study selection**

^a^Data were collected from Korean GlomeruloNEphritis sTudy group (KoGNET) database

Abbreviations: GN, glomerulonephritis; IgAN, IgA nephropathy; HSP, Henoch-schönlein purpura; LM, light microscope.

**
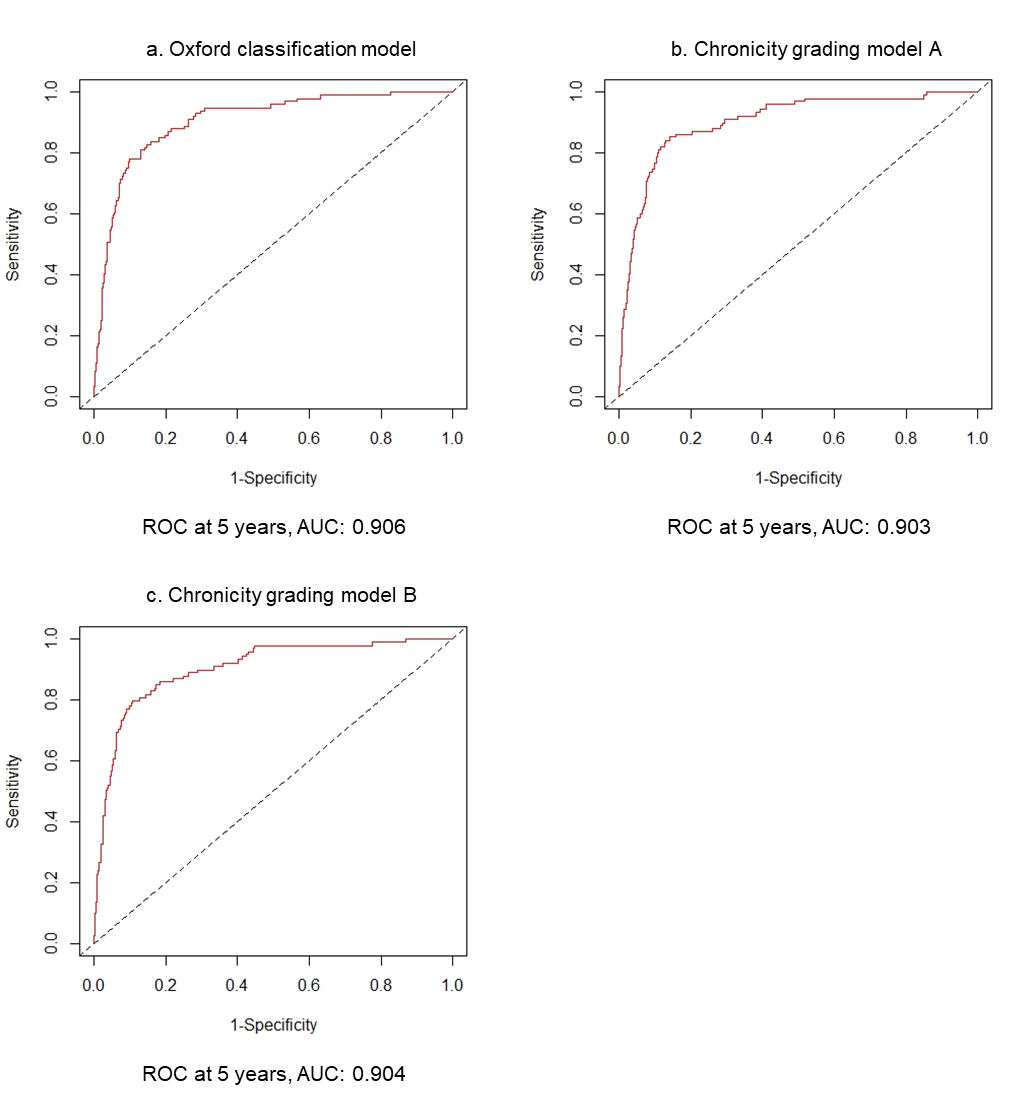
**

**Supplementary Figure 2. ROC curves for predicting ESRD progression at 5 years**

Abbreviations: ROC, receiver operating characteristic; AUC, area under the ROC curve.

**
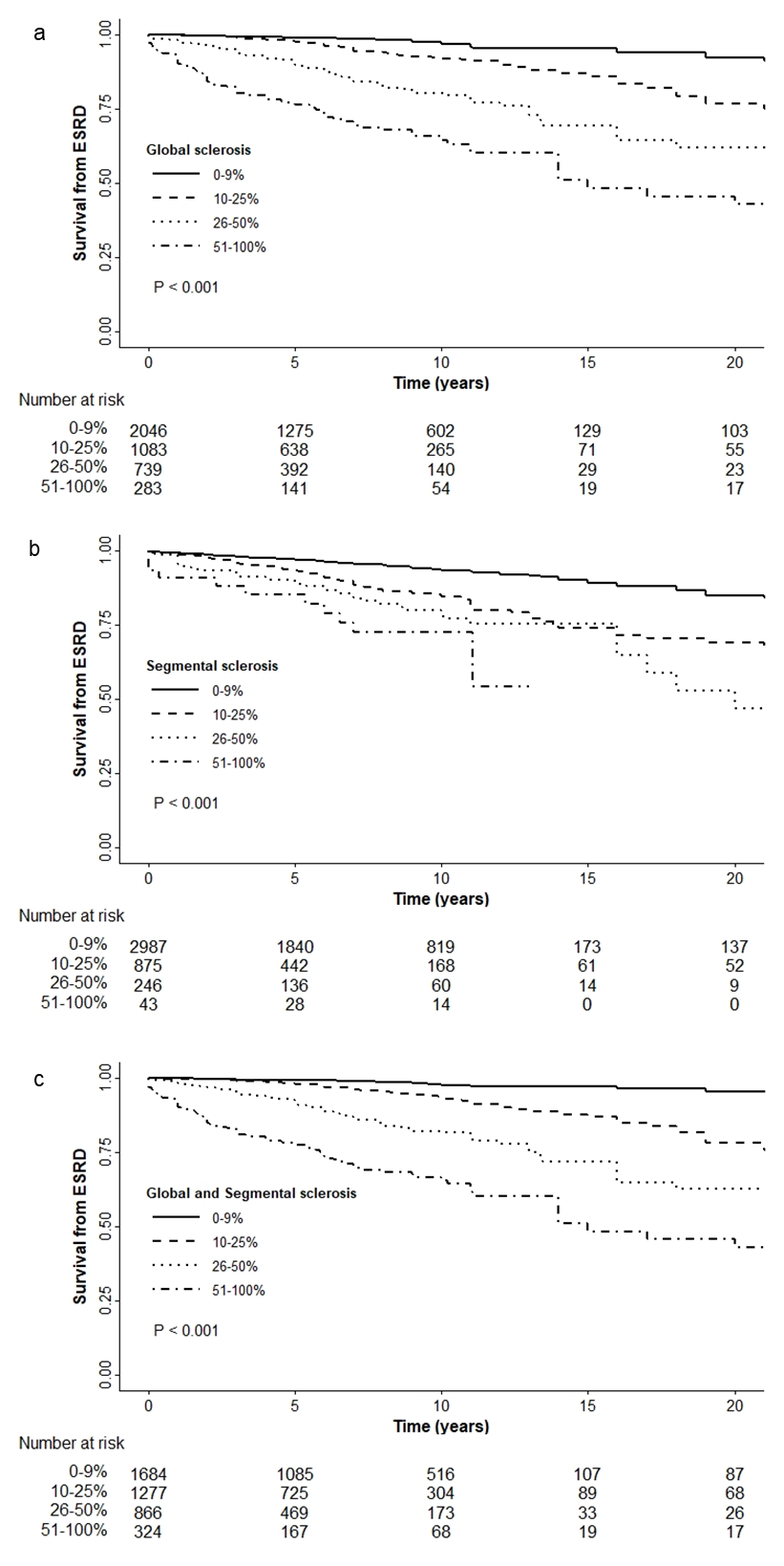
**

**Supplementary Figure 3. Renal outcome with glomerulosclerosis lesions. (a) Global sclerosis with ESRD progression. (b) Segmental sclerosis with ESRD progression. (c) Combined global and segmental sclerosis with ESRD progression**
